# Evaluating the impact of chemotherapy-induced nausea and vomiting on daily functioning in patients receiving dexamethasone-sparing antiemetic regimens with NEPA (netupitant/palonosetron) in the cisplatin setting: results from a randomized phase 3 study

**DOI:** 10.1186/s12885-022-10018-3

**Published:** 2022-08-24

**Authors:** Luigi Celio, Diego Cortinovis, Alessio Aligi Cogoni, Luigi Cavanna, Olga Martelli, Simona Carnio, Elena Collovà, Federica Bertolini, Fausto Petrelli, Alessandra Cassano, Rita Chiari, Francesca Zanelli, Salvatore Pisconti, Isabella Vittimberga, Antonietta Letizia, Andrea Misino, Angela Gernone, Erminio Bonizzoni, Sara Pilotto, Sabino De Placido, Emilio Bria

**Affiliations:** 1 Medical Oncology Unit, ASST del Garda, Desenzano del Garda Hospital, Località Montecroce 1, 25015 Desenzano del Garda, BS Italy; 2 Medical Oncology Department, ASST Monza San Gerardo Hospital, Monza, Italy; 3 Medical Oncology Department, Azienda Ospedaliero-Universitaria di Sassari, Sassari, Italy; 4 Oncology Department, Azienda Ospedaliera di Piacenza, Piacenza, Italy; 5 Medical Oncology, ASL Frosinone, Frosinone, Italy; 6 Department of Oncology, San Luigi Gonzaga Hospital, University of Turin, Orbassano, Turin, Italy; 7 Cancer Centre Department – Oncology Unit, ASST Ovest Milanese – Legnano Hospital, Legnano, Milan, Italy; 8 Department of Oncology and Hematology, AOU Policlinico di Modena, Modena, Italy; 9 Medical Oncology Unit, ASST Bergamo Ovest, Treviglio, Bergamo, Italy; 10 Comprehensive Cancer Center, Fondazione Policlinico Universitario Agostino Gemelli, IRCCS, Rome, Italy; 11 Medical Oncology, Università Cattolica del Sacro Cuore, Rome, Italy; 12 Oncology Unit, AULSS6 Euganea, Padova, Italy; 13 Medical Oncology Unit, IRCCS Santa Maria Nuova, Reggio Emilia, Italy; 14 Medical Oncology Department, San Giuseppe Moscati Hospital, Statte, Taranto, Italy; 15 Department of Oncology, ASST Lecco, Lecco, Italy; 16 Department of Pneumology and Oncology, AORN dei Colli-Ospedale Monaldi, Naples, Italy; 17 Medical Oncology, Clinical Cancer Center “Giovanni Paolo II” – IRCCS, Bari, Italy; 18 Medical Oncology Unit, University of Bari, Policlinico di Bari, Bari, Italy; 19 Department of Clinical Science and Community, Section of Medical Statistics, Biometry and Epidemiology “G.A. Maccacaro”, Faculty of Medicine and Surgery, University of Milan, Milan, Italy; 20 Section of Oncology, Department of Medicine, University and Hospital Trust of Verona, Verona, Italy; 21 Clinical Medicine and Surgery Department, University of Naples “Federico II”, Naples, Italy

**Keywords:** Cisplatin, Dexamethasone, NEPA, Quality of life, Functional living index-Emesis, Chemotherapy-induced nausea and vomiting (CINV)

## Abstract

**Background:**

The non-inferiority of dexamethasone (DEX) on day 1, with or without low-dose DEX on days 2 and 3, combined with oral NEPA (netupitant/palonosetron), compared with the guideline-consistent use of DEX was demonstrated in cisplatin. Here, we complete the analysis by assessing the impact of emesis on daily lives of patients receiving DEX-sparing regimens using the Functional Living Index-Emesis (FLIE).

**Methods:**

Chemotherapy-naïve patients undergoing cisplatin (≥70 mg/m^2^), were given NEPA and DEX (12 mg) on day 1 and randomized to receive either 1) no further DEX (DEX1), 2) oral DEX (4 mg daily) on days 2–3 (DEX3), or 3) DEX (4 mg twice daily) on days 2–4 (DEX4; control). Patients completed the FLIE questionnaire on day 6 of cycle 1. Endpoints included the FLIE nausea domain, vomiting domain, and overall combined domain scores, as well as the proportion of patients with no impact on daily life (NIDL; overall score > 108). This was a protocol-planned analysis.

**Results:**

In the DEX1 group, no significant differences were observed in the FLIE nausea score (48.9 [±1.8; SE] vs. 53.7 [±1.5]), vomiting score (56.6 [±1.4] vs. 58.7 [±0.8]) and overall score (105.6 [±2.8] vs.112.4 [±1.9]) versus DEX4 control; similar results were observed in the DEX3 group for nausea score (49.6 [±1.7]), vomiting score (58.2 [±1]) and overall score (107.8 [±2.4]) versus control. There were no significant between-group differences in the proportion of patients reporting NIDL.

**Conclusion:**

Reducing DEX, when administered with NEPA, does not seem to adversely impact the daily functioning in patients undergoing cisplatin.

**Trial registration:**

ClinicalTrials.govNCT04201769. Registration date: 17/12/2019 - Retrospectively registered.

## Background

Chemotherapy-induced nausea and vomiting (CINV) has a deleterious effect on health-related quality of life (QoL) but prevention of symptoms can impact QoL positively [1–4]. Substantial progress in antiemetic research has led to the development of highly effective drugs for the control of CINV occurring in the acute (within 24 h following chemotherapy administration) and delayed (days 2–5 after chemotherapy) phases. Currently, the major international guidelines recommend a triple combination of a neurokinin-1 receptor antagonist (NK-1 RA), a 5-hydroxytryptamine-3 (5-HT_3_) RA and 4-days of dexamethasone (DEX), with or without olanzapine, for the prevention of CINV caused by highly emetogenic chemotherapy (HEC) containing cisplatin [5, 6]. Several studies have demonstrated that adherence to guideline recommendations in clinical practice is largely suboptimal, and this can result in uncontrolled CINV [7–9]. Therefore, there is a need to evaluate whether different treatment strategies can offer high protection with simplified antiemetic regimens in order to improve physician adherence to guideline recommendations, as well as patient compliance to antiemetic prophylaxis.

DEX used for the prevention of CINV may be contraindicated in some instances such as patients who experience DEX-related side effects or in those with pre-existing conditions that may be exacerbated by corticosteroids [10–13]. Therefore, there has been growing interest in minimizing DEX dose/frequency in each cycle of chemotherapy [14–16]. We designed a randomized, non-inferiority study to assess whether two DEX-sparing regimens used with NEPA, a fixed-dose combination of the NK-1RA, netupitant, and the 5-HT_3_ RA, palonosetron, might provide the opportunity to reduce the total corticosteroid dose while maintaining the same degree of CINV control in patients undergoing common high-doses of cisplatin. Efficacy results of the parent study are reported in full elsewhere [17]. The study demonstrated non-inferiority of the DEX-sparing regimens to the standard 4-day DEX regimen for the primary endpoint of complete response (CR; no emesis and no rescue medication) during the overall phase post-chemotherapy.

In the current analysis, we explore the functional impact of DEX-sparing regimens on health-related QoL by using the Functional Living Index-Emesis (FLIE), a validated nausea- and vomiting-specific patient-reported outcome measure. Use of the FLIE has previously demonstrated that effective antiemetic prophylaxis reduces the negative impact of CINV on daily life activities [18].

## Methods

The present analysis deals with a pre-specified secondary endpoint in an investigator-initiated, phase 3b, open-label, multicenter, randomized, three-arm study that aimed to evaluate the non-inferiority of two DEX-sparing regimens when combined with oral NEPA versus the guideline-consistent DEX regimen in patients receiving cisplatin-containing chemotherapy. This study was registered on ClinicalTrials.gov (identifier NCT04201769) on 17/12/2019 and on the European Union Clinical Trials Register on 08/06/2016 (EudraCT number 2015–005704-29). The phase 3 study was conducted in compliance with the International Conference on Harmonization and Good Clinical Practice guidelines. Detailed methods and results regarding the prevention of CINV were previously reported [17].

In the parent study, eligible patients were > 18 years of age with a confirmed diagnosis of non-small cell lung cancer (NSCLC), chemotherapy-naive and scheduled to receive their first course of cisplatin (≥70 mg/m^2^)-based chemotherapy. Patients were excluded if they were scheduled to receive either concurrent chemo-radiation therapy for NSCLC or radiation therapy to the abdomen or pelvis within 1 week before chemotherapy initiation, had symptomatic brain metastases, had contraindications for corticosteroid use, had routine use of corticosteroids or any other agent with antiemetic potential, or had nausea or vomiting within 24 h before chemotherapy initiation.

On the 5 days following initiation of cisplatin, patients used a diary to record the occurrence of emetic episodes, any use of rescue medication, and daily ratings of nausea severity using a categorical Likert scale. The primary endpoint of the parent study was the proportion of patients achieving complete response (CR; defined as no emetic episode and no use of rescue medication) in the overall phase (0 to 120 h from the initiation of cisplatin).

### Treatment

Patients were given NEPA and DEX (12 mg intravenously) and randomized (1:1:1) to receive either 1) no further DEX (DEX1), 2) oral DEX (4 mg daily) on days 2–3 (DEX3), or 3) oral DEX (4 mg twice daily) on days 2–4 (DEX4; the reference group). Patients were allowed to take rescue medication throughout the study period for nausea or vomiting, if necessary. The choice of recommended rescue medicine was either DEX or metoclopramide and was at the discretion of each investigator.

### FLIE measurement and scoring

The FLIE questionnaire comprised of two domains (nausea and vomiting) with 9 identical items in each domain [19]. Patients completed the FLIE questionnaire on day 6, assessing the impact of CINV on their daily functioning during the 120 h after chemotherapy administration. The average score for each domain was summed and transformed using a pre-specified scoring procedure, which allowed for a minimum domain score of 9 and a maximum of 63. FLIE responses were summed to determine the overall combined score (range 18–126) and the nausea domain and vomiting domain scores (range 9–63) [19]. A higher score reflects less impact on daily life. An overall combined domain score of more than 108 (i.e., scores more than 54 for each domain) has been shown to be associated with no impact on daily life (NIDL). If more than four of nine questions were missed in a domain, subtotal and overall scores were considered missing [19].

### Statistical analysis

Analyses for the FLIE were performed using a modified per-protocol population (mPP) that included all randomized patients who were compliant with the study protocol and had a valid FLIE questionnaire obtained on day 6 of cycle 1. The mPP population was also used in re-analyzing the primary efficacy endpoint. Analyses of treatment group differences were completed separately for the FLIE nausea domain, vomiting domain, and overall combined scores. The Confidence Interval (CI) Inclusion Approach was used for comparison of group means, while differences in the proportion of patients with NIDL between treatment groups were analyzed using Fisher’s exact test. Two-sided *P-*values of < 0.05 were considered to be statistically significant and were not adjusted for multiplicity. Descriptive statistics were used to summarize percentage of patients reporting NIDL and FLIE overall combined score by acute and/or delayed antiemetic control.

## Results

A total of 228 patients were included in the per-protocol population for the parent study [17]. No FLIE questionnaires could be obtained from 14 patients (six patients in the DEX1 arm, four in the DEX3 arm, and four in the DEX4 arm). Questionnaires from 2 further patients (one in each of the DEX3 and DEX4 arms) had to be excluded from the analysis because of incomplete data. Hence, in the mPP population, 212 patients were assessable for the present analysis. Demographic data and baseline patient characteristics by treatment group are shown in Table 1. The majority of patients evaluated (67%) were male. All three treatment groups were comparable regarding other baseline characteristics.

**Table 1:** Baseline demographics and patient characteristics of the mPP population

Characteristic	NEPA + DEX1 (***n*** = 70)	NEPA + DEX3 (***n*** = 71)	NEPA + DEX4 (***n*** = 71)
Age (years)			
mean (SD)	64.2 (7.3)	62.4 (8.3)	63.4 (8.0)
median (min-max)	67 (44–79)	63 (34–77)	64 (40–76)
Gender, n (%)			
Male	51 (72.9)	41 (57.7)	49 (69.1)
Female	21 (27.1)	30 (42.3)	22 (30.9)
Alcohol consumption, n (%)			
Never	51 (72.9)	41 (57.8)	44 (62)
Every day	19 (27.1)	29 (40.8)	26 (36.6)
Unknown	–	1 (1.4)	1 (1.4)
Motion sickness, n (%)			
Yes	9 (12.9)	10 (14.1)	7 (9.9)
No	57 (81.4)	60 (84.5)	61 (85.9)
Unknown	4 (5.7)	1 (1.4)	3 (4.2)

*Abbreviations*: *mPP* modified per-protocol, *NEPA* a fixed combination of netupitant and palonosetron, *DEX1* dexamethasone day 1, *DEX3* dexamethasone day 1 to 3, *DEX4* dexamethasone day 1 to 4, *SD* standard deviation

In the mPP population, the proportion of patients with a CR in the overall phase was comparable across groups: 74.3 and 76.1% in the DEX-sparing groups and 76.1% in the standard 4-day DEX group.

### Impact of nausea and vomiting on patient’s daily life

**Fig. 1:**
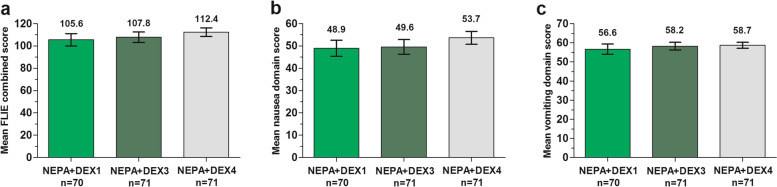
FLIE outcome assessments. **a** Mean FLIE overall combined score (range 18–126), **b** mean nausea domain score (range 9–63), **c** mean vomiting domain score (range 9–63). Error bars represent 95% confidence interval. *Abbreviations:* FLIE, Functional Living Index-Emesis; NEPA, a fixed combination of netupitant and palonosetron; DEX1, dexamethasone day 1; DEX3, dexamethasone day 1 to 3; DEX4, dexamethasone day 1 to 4

The mean FLIE overall combined scores in both DEX-sparing groups were slightly lower than that in the reference group, but differences were not statistically significant (DEX1 vs. DEX4: 95% CI for difference, − 13.5 to 0.05; *P* = 0.06; DEX3 vs. DEX4: 95% CI for difference, − 10.6 to 1.5; *P* = 0.15; Fig. 1a). The mean FLIE scores for the nausea domain slightly favored the reference group, but no statistically significant differences between groups were observed (DEX1 vs. DEX4: 95% CI for difference, − 9.3 to 0.01; *P* = 0.07; DEX3 vs. DEX4: 95% CI for difference, − 8.4 to 0.3; *P* = 0.09; Fig. 1b). The mean FLIE scores for the vomiting domain in both DEX-sparing groups were comparable to that in the reference group (DEX1 vs. DEX4: 95% CI for difference, − 5.2 to 1; *P* = 0.55; DEX3 vs. DEX4: 95% CI for difference, − 3 to 2.1; *P* = 0.87; Fig. 1c).

**Fig. 2:**
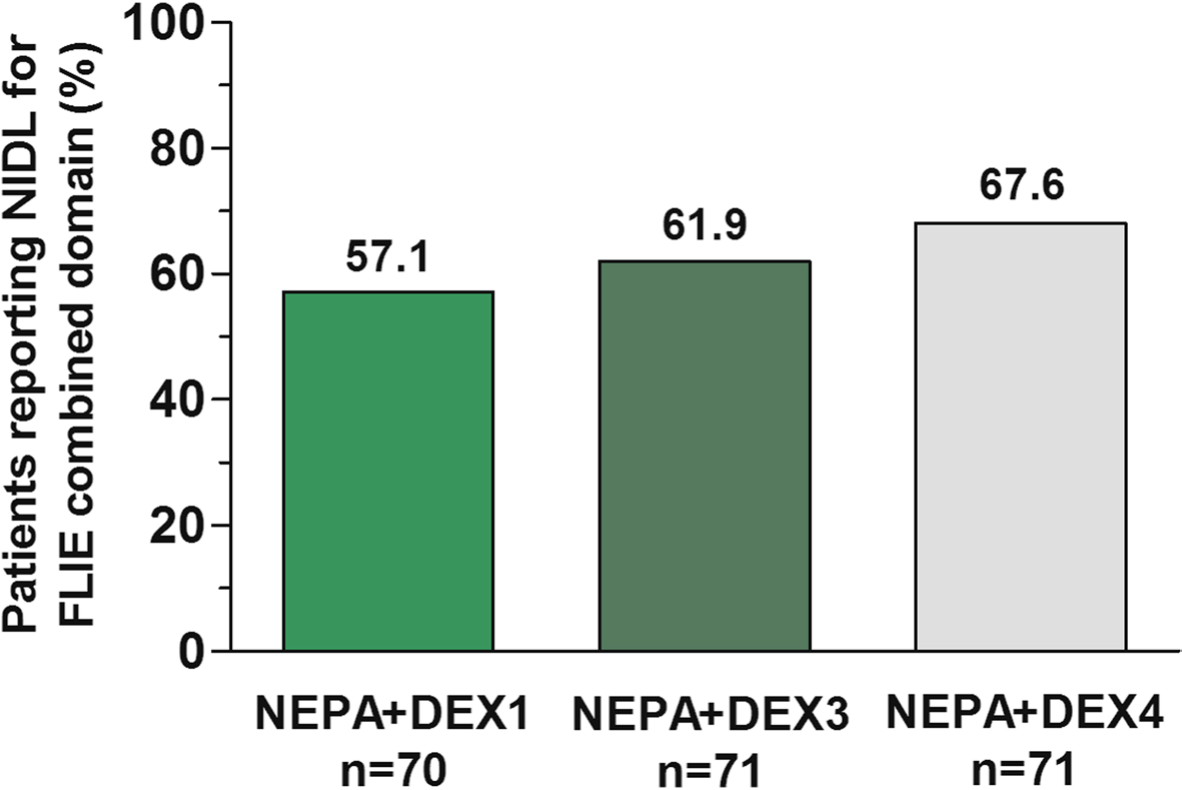
Percentage of patients with NIDL for FLIE overall combined score. *Abbreviations:* FLIE, Functional living Index-Emesis; NIDL, no impact on daily life; NEPA, a fixed combination of netupitant and palonosetron; DEX1, dexamethasone day 1; DEX3, dexamethasone day 1 to 3; DEX4, dexamethasone day 1 to 4

The proportion of patients reporting NIDL for the overall combined domain in both DEX-sparing groups was lower than that in the reference group, but differences were not statistically significant (DEX1 vs. DEX4: 95% CI for difference, − 26.4 to 5.4%; *P* = 0.23; DEX3 vs. DEX4: 95% CI for difference, − 21.3 to 10.1%; *P* = 0.60; Fig. 2).

### Impact of CINV control on patient’s daily life

Table 2 reports the proportion of patients reporting NIDL relative to whether they experienced acute or delayed CR. The responder analysis showed that the FLIE data for the overall combined domain were consistent with the primary efficacy endpoint of CR, also confirming the discriminant validity of the FLIE. The proportions of patients achieving overall (acute + delayed) CR also reported NIDL and were similar across treatment groups; consistently, among the patients who did not achieve a CR during the acute and delayed phases, the majority reported a negative impact on daily living. Comparisons between groups also showed that patients achieving CR during the acute and delayed phases in each of the DEX-sparing groups reported, on average, FLIE overall combined scores comparable to that in the DEX4 group.

**Table 2:** Proportion of patients reporting NIDL and overall combined FLIE score relative to experiencing complete response

Treatment group	CR			Patients reporting NIDL		FLIE overall combined score	
	Acute	Delayed	No. of patients	***N***	%	Mean (SD)	Median (range)
NEPA+DEX1	Yes	Yes	52	38	73.1	113.1 (16.6)	118.2 (28.7–126)
	Yes	No	13	2	15.4	85.6 (26.8)	89.6 (23.6–120.5)
	No	Yes	0	–	–	–	–
	No	No	5	0	0	79.1 (30.5)	93.5 (31–102.2)
NEPA+DEX3	Yes	Yes	54	41	75.9	112.6 (17.8)	118.4 (31.1–126)
	Yes	No	10	3	30	88.6 (23.1)	89.9 (46.9–115.7)
	No	Yes	0	–	–	–	–
	No	No	7	0	0	98.7 (10.9)	102.6 (74.8–105.9)
NEPA+DEX4	Yes	Yes	54	43	79.6	117 (8.4)	119.2 (99–126)
	Yes	No	10	3	30	91.6 (29)	95.6 (27–124.2)
	No	Yes	0	–	–	–	–
	No	No	7	2	28.6	106.2 (16.9)	114.2 (80.1–121.1)

Acute refers to 0–24 h after chemotherapy and delayed refers to 25–120 h after chemotherapy. NIDL is based on FLIE overall combined score. The FLIE questionnaire was completed on day 6 post-chemotherapy.

*Abbreviations: SD* standard deviation, *NIDL* no impact on daily life, *FLIE* Functional Living Index-Emesis, *CR* complete response (no emetic episode and no use of rescue medication), *NEPA* a fixed combination of netupitant and palonosetron, *DEX1* dexamethasone day 1, *DEX3* dexamethasone day 1 to 3, *DEX4* dexamethasone day 1 to 4

## Discussion

For the first time, we used the validated FLIE questionnaire to assess the consequences of CINV on health-related QoL during the cycle 1 of cisplatin-based HEC in patients who were randomized to receive DEX-sparing regimens or a guideline-consistent (standard of care) antiemetic prophylactic regimen [17]. Overall, the additional parameters analyzed in this analysis support the non-inferiority efficacy outcome of the parent study, providing evidence that tailoring DEX dosing to reduce exposure is not associated with a significant loss in health-related QoL in patients undergoing cisplatin. This is consistent with the study by Ito et al. in which no statistically significant difference was observed in mean scores of patients’ global health status, as assessed by the EORTC Quality of Life Questionnaire C30, between a DEX-sparing regimen and a 3-day DEX regimen in patients undergoing HEC [15]. However, since the vast majority of patients were women treated with the combination of an anthracycline and cyclophosphamide, post-hoc analyses failed to demonstrate the non-inferiority hypothesis in patients receiving cisplatin.

In the mPP population, we observed comparable rates of CR across groups in the overall phase. Therefore, the present results are consistent with the findings from the parent study [17]. The mean overall combined domain FLIE scores indicated that the patients in both DEX-sparing groups, on average, did not experience CINV that negatively impacted their lives. The mean vomiting domain scores were comparable across groups, also indicating a lack of negative impact. As expected, the mean nausea domain scores were lower than those for the vomiting domain in each treatment group, indicating that some patients experienced more impact from nausea. Also, the lower scores for nausea were aligned with the proportion of patients reporting NIDL for the FLIE overall combined domain in both experimental groups. It should be noted that in the responder analysis, the patients in both DEX-sparing groups who did not achieve CR during the overall phase, on average, reported lower scores for the overall combined domain than those observed among the patients who did not experience CR in the reference group. While this contributed to lower the mean overall combined domain FLIE scores in both DEX-sparing groups, there was no meaningful impact on the proportion of patients reporting NIDL in the two treatment groups. In addition, the responder analysis revealed that in the reference group, two patients who reported NIDL did not achieve CR (i.e., experienced CINV) during the acute and delayed phases. Interestingly, one of these patients took rescue medication due to mild nausea. This finding is consistent with the literature data which indicate that a large number of patients who report taking rescue medication have mild or no nausea and no emesis [20]. Considering this, it is likely that this patient would report NIDL for the overall combined domain regardless of the DEX dosing regimen administered.

It is well known that prevention of nausea, especially delayed nausea, continues to present a treatment challenge in the management of CINV [21]. Therefore, clinicians should keep in mind some aspects when evaluating the impact of the DEX-sparing regimens on health-related QoL in the cisplatin setting. Firstly, while vomiting is a time point event, the feeling of nausea is prolonged in time and therefore may have more impact on daily functioning [22]. In light of this, the FLIE results in both DEX-sparing groups are reassuring as they were observed in patients at very high risk for delayed nausea due to treatment with high-dose (≥70 mg/m^2^) cisplatin. This view is supported by the evidence from a prospective observational study evaluating the impact of delayed CINV on daily life activities. In this analysis, patients who experienced delayed but not acute nausea were more likely to report a detrimental effect on daily functioning than patients who had only acute nausea [2]. Secondly, in the present analysis the FLIE results corroborate the efficacy outcome of CR which allows only indirect assessment of nausea by the term “no rescue medication”, a surrogate marker for no nausea or only mild nausea [21]. The responder analysis highlighted that a similar proportion of patients achieving overall (acute + delayed) CR in each treatment group reported NIDL for the overall combined domain, while very few patients who only achieved acute CR reported NIDL on day 6 regardless of the DEX dosing regimen. Finally, the present analysis suggests that a negative impact on daily living may occur in patients treated with DEX-sparing regimens experiencing delayed CINV. In light of this, for patients who undergo cisplatin and have an inherent high-risk for developing CINV, clinicians should opt for a 4-day DEX regimen to avoid potential poor protection against delayed nausea, if they anticipate no side effects related to multiple days of the steroid [10–13]. An alternative option would be the upfront addition of olanzapine to the DEX-sparing strategy [5, 6]. Interestingly, a recent randomized study showed a significant improvement in control of delayed nausea for patients who received low-dose olanzapine combined with palonosetron, 3-day aprepitant, and 4-day DEX versus those who received placebo plus triplet-combination prophylaxis during treatment with cisplatin [23]. Adding low-dose olanzapine to NEPA with single-dose DEX might provide the opportunity to achieve both incremental antiemetic benefit and improved tolerability in this challenging setting of CINV, while greatly simplifying the complex four-drug regimen. Randomized studies comparing olanzapine with or without the DEX-sparing strategy are ongoing in the cisplatin setting [24].

This analysis has some limitations. Since the analysis deals with a pre-specified secondary endpoint of the parent study, the current findings should be considered to be only exploratory. Additionally, our analyses did not control for baseline FLIE scores. We did not use the FLIE questionnaire to assess changes from the baseline in patient’s functional status following treatment; conversely, the aim was to assess the between-group differences in the proportion of patients reporting NIDL for overall combined domain [3, 18]. We administered the FLIE questionnaire on day 6, a period that was judged to be adequate on the basis of results in the literature [2] and validated in a clinical trial sample [19]. Furthermore, the 5-day period is expected to include most CINV-related events without a relevant level of recall bias [2, 3]. Although the study design did not include matching placebo medication, this is not expected to negatively impact the FLIE scores in the DEX-sparing groups.

## Conclusion

The present analysis provides evidence that in patients undergoing high-dose cisplatin a simplified antiemetic prophylactic regimen of NEPA with single-dose DEX, both administered before chemotherapy initiation, does not seem to adversely affect patient functioning.

## Data Availability

The datasets analyzed during the present study will be available on reasonable request.

## Abbreviations

5-HT3: 5-hydroxytryptamine-3
CINV: Chemotherapy-induced nausea and vomiting
CR: Complete response
DEX: Dexamethasone
DEX1: Dexamethasone day 1
DEX3: Dexamethasone day 1 to 3
DEX4: Dexamethasone day 1 to 4
EORTC: European Organization for Research and Treatment of Cancer
FLIE: Functional Living Index-Emesis
HEC: Highly emetogenic chemotherapy
mPP: Modified per-protocol population
NEPA: Netupitant palonosetron fixed combination
NIDL: No impact on daily living
NK1: Neurokinin-1
NSCLC: Non-small cell lung cancer
RA: Receptor antagonist
QoL: Quality of life
SD: Standard deviation
